# From anticipation to impulsivity in Parkinson’s disease

**DOI:** 10.1038/s41531-022-00393-w

**Published:** 2022-10-03

**Authors:** Bertrand Degos, Pierre Pouget, Marcus Missal

**Affiliations:** 1 Department of Neurology, Avicenne University Hospital, AP-HP, Sorbonne Paris Nord University, NS-PARK/FCRIN network, 93009 Bobigny, France; 2 Dynamics and Pathophysiology of Neuronal Networks Team, Center for Interdisciplinary Research in Biology, Collège de France, CNRS UMR7241/INSERM U1050, Université PSL, Paris, France; 3 ICM, CNRS, INSERM, Université Pierre et Marie Curie, Hôpital de la Salpêtrière, Paris, France; 4 Institute of Neurosciences (IONS), Cognition and System (COSY), Université catholique de Louvain, 53 av Mounier, B1.53. 4 COSY, 1200 Brussels, Belgium

**Keywords:** Parkinson's disease, Oculomotor system

## Abstract

Anticipatory actions require to keep track of elapsed time and inhibitory control. These cognitive functions could be impacted in Parkinson’s disease (iPD). To test this hypothesis, a saccadic reaction time task was used where a visual warning stimulus (WS) predicted the occurrence of an imperative one (IS) appearing after a short delay. In the implicit condition, subjects were not informed about the duration of the delay, disfavoring anticipatory behavior but leaving inhibitory control unaltered. In the explicit condition, delay duration was cued. This should favor anticipatory behavior and perhaps alter inhibitory control. This hypothesis was tested in controls (*N* = 18) and age-matched iPD patients (*N* = 20; ON and OFF L-DOPA). We found that the latency distribution of saccades before the IS was bimodal. The 1^st^ mode weakly depended on temporal information and was more prominent in iPD. Saccades in this mode were premature and could result of a lack of inhibition. The 2^nd^ mode covaried with cued duration suggesting that these movements were genuine anticipatory saccades. The explicit condition increased the probability of anticipatory saccades before the IS in controls and iPD_ON_ but not iPD_OFF_ patients. Furthermore, in iPD patients the probability of sequences of 1^st^ mode premature responses increased. In conclusion, the triggering of a premature saccade or the initiation of a controlled anticipatory one could be conceptualized as the output of two independent stochastic processes. Altered time perception and increased motor impulsivity could alter the balance between these two processes in favor of the latter in iPD, particularly OFF L-Dopa.

## Introduction

The initiation of an anticipatory action requires to keep track of elapsed time. For instance, a precise anticipation of the moment when a traffic light will turn green requires to keep track of time during the period when it was red. Although there is no sensory pathway for the ‘perception’ of time, dysfunction of the basal ganglia (BG) in Parkinson’s disease (iPD^1^) perturbs the ability to estimate, produce or reproduce the duration of a sensory stimulus, e.g., a tone^2–26^. This distorted representation of time could partly explain the reduced ability to initiate predictive or anticipatory eye movements observed in iPD^27–30^.

However, the initiation of an anticipatory eye movement at the right time also requires the ability to refrain from responding too early by exerting inhibitory control. Inhibitory control is an executive function allowing to prioritize actions and execute them in an orderly sequence^31^. In general, unwanted movements are inhibited during motor preparation to allow the execution of only the selected one^32^. Deficits of inhibitory control have been repeatedly observed in iPD patients in tasks requiring a cognitive effort and/or inhibition of prepotent responses^33–36^. In general, reduced inhibitory control could be one of the causes of impulsivity that is usually defined as the tendency to act without forethought or execute actions prematurely^37–40^. Impulsivity is a multifactorial construct but some aspects of it could be increased in iPD, even in the absence of an observable impulse control disorder^41,42^. The motor consequences of increased impulsivity can be precisely evaluated using eye movements. Indeed, the saccadic system is under strong inhibitory control unless a saccade is planned to explore the environment or catch with the eye an object of interest. Premotor neurons for saccadic eye movement are normally under strong inhibitory control and oculomotor impulsivity could occur if this ‘gate’ is unstable^43^.

Typically, in a saccadic reaction time experiment, a warning stimulus (WS; e.g., a flashed visual stimulus on a computer screen) is followed by an imperative stimulus (IS; e.g., a visual target) after a variable delay (or foreperiod, FP^44^). Experimental subjects are instructed to wait for the appearance of the IS before making an eye movement. Nevertheless, saccadic eye movements often occur before the IS. In the present study, saccadic eye movements occurring before the IS will be qualified as ‘early’. Early saccades could reach up to 14% of all responses in healthy subjects and up to 31% in Major Depression Disorder^45^. They could be caused either by a lack of inhibitory control evoking a ‘premature’ saccade or result from a genuine attempt to predict the IS in the temporal domain evoking an ‘anticipatory’ saccade. Therefore, temporal information could allow to differentiate these two categories of movements and evaluate their relative occurrence in iPD patients compared with healthy controls. Indeed, if experimental subjects are not informed about the duration of the FP, the timing of the IS is an ‘implicit’ variable that should not favor anticipation. However, premature movements should be unaffected given that they could be due to an unintentional fluctuation of inhibitory control. In contrast, if experimental subjects are informed about the duration of the expected FP with a visual cue, then they could explicitly try to use that prior information to predict the occurrence of the IS and initiate an anticipatory saccade^46^.

In the present study, we hypothesized that timing context, implicit or explicit, could differentiate oculomotor impulsivity from anticipation. Furthermore, we suggest that iPD could cause both a reduction of inhibitory control resulting in more premature saccades and alter the timing of anticipatory eye movements. Analysis of average reaction times will be used to test these hypotheses and allow a statistical comparison between groups. In addition, a Markov process analysis will be used to determine the context triggering a premature movement or a genuine anticipatory saccade in controls and patients, ON or OFF L-DOPA medication.

## Results

### Demographic and clinical characteristics of participants

Eighteen healthy control subjects (2 females, 16 males) and twenty Parkinson’s disease patients (amongst which 3 females) took part to the present study (see Table 1). All the 20 PD patients presented the idiopathic form of the disease (referred to as ‘iPD’). Between groups, there was no statistically significant difference of age (F[1, 36] = 0.000; *P* = 0.988; NS), Starkstein score (F[1, 35] = 2.056; *P* = 0.160; NS) or MoCA score (F[1, 36] = 1.341; *P* = 0.255; NS). Education level was also compared between controls and iPD patients. Subjects and patients were scored according to the maximum level of education attained (High School, score ‘0’; higher than High School, score ‘1’). For practical reasons, this information could be collected only for 13 control subjects and 15 iPD patients. A chi-square test revealed no statistically significant difference of education score between groups (χ^2^ [1, 28] = 0.444, *p* = 0.505).

**Table 1:** Demographics and clinical measures.

	Controls (*n* = 18)	iPD (*n* = 20)
Age (years)	58.0 ± 1.7	58.6 ± 1.7
Age onset (years)	na	53.6 ± 1.9
Duration (years)	na	4.9 ± 0.7
Hoehn and Yahr	na	1.8 ± 0.1
Starkstein ON (/42)	8.1 ± 0.7	11.4 ± 1.1
Starkstein OFF	na	11.4 ± 1.3
UPDRS III ON	na	13.6 ± 1.6
UPDRS III OFF	na	23.8 ± 2.2
MoCA	28.6 ± 0.3	28.4 ± 0.6
L-Dopa equi (mg)	na	523 ± 54.9

*iPD* idiopathic Parkinson’s disease patients; *L-Dopa* (mg) Levodopa equivalence in milligrams. For the Starkstein score ON Levodopa, *n* = 19 in the iPD group. For the Starkstein score OFF Levodopa, *n* = 14 in the iPD group.

### Macro level analysis of saccadic latencies

**Fig. 1:**
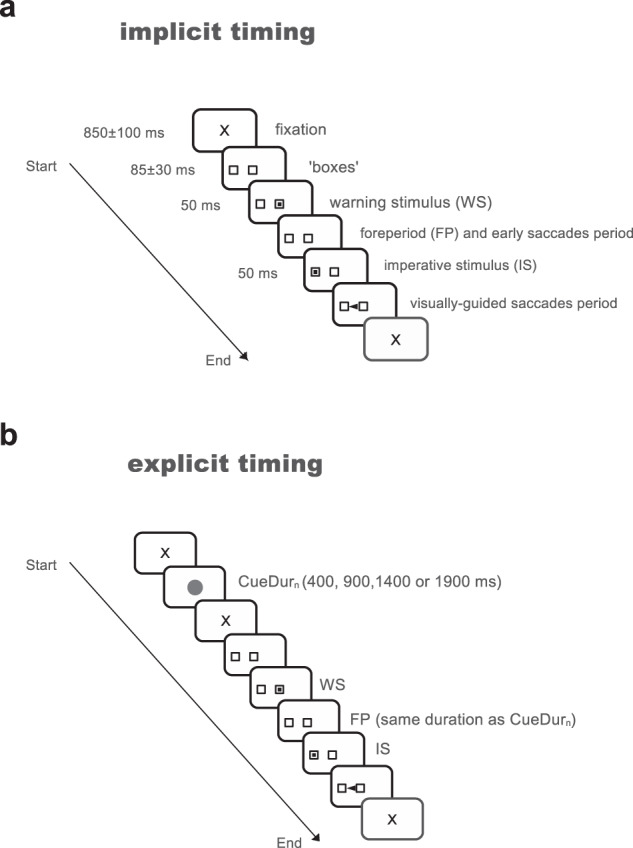
Schematic drawing of the different experimental conditions. Subjects were facing a computer screen on which stimuli were displayed. **a** Implicit timing condition: the trial started with the appearance of a fixation cross for a randomized duration followed by the appearance of two empty ‘boxes’, one at the center of the screen and a second one at a 9-deg eccentric position. After the appearance of the two boxes, a target was briefly presented in the central one for 50 ms. Disappearance of the central target marked the beginning of the foreperiod (FP) that could last either 400, 900, 1400, or 1900 ms. At the end of this delay period, a target appeared for 50 ms in the eccentric box. The instruction given to subjects was to make a visually-guided eye saccade to the eccentric target as soon as possible. However, ‘early’ saccades occurred frequently during the FP. **b** Explicit timing condition: the trial started with a fixation cross (same duration as in implicit condition), followed by the cue period when a red disk was presented on the screen for one of the four durations selected randomly. A short fixation period followed and the two empty boxes appeared on the screen. The end of the trial was similar as in the implicit case. Cue duration allowed subjects to predict upcoming FP duration.

Figure 1 describes the experimental paradigms used in the implicit and explicit conditions (see Methods for more details). The analysis presented here will concentrate on saccadic eye movements that occurred before the imperative stimulus (IS) in both the implicit and explicit conditions. These saccades will be collectively referred to as ‘early saccades’ in a first approach. All trials that did not belong to either the early or visual categories were considered as failed (see Table 2).

**Table 2:** Summary.

Group_condition_	Number of subjects	Failed trials	Visually-guided saccades	Early saccades
CONT_IMP_	18	20 ± 4% *n* = 1059	74 ± 4% *n* = 4503	6 ± 1% *n* = 398
CONT_EXP_	18	19 ± 4% *n* = 564	63 ± 5% *n* = 2000	18 ± 5% *n* = 621
iPD_ON_IMP_	20	45 ± 5% *n* = 1920	46 ± 5% *n* = 1951	9 ± 2% *n* = 392
iPD_ON_EXP_	20	38 ± 5% *n* = 963	44 ± 6% *n* = 947	20 ± 4% *n* = 471
iPD_OFF_IMP_	20	40 ± 4% *n* = 1633	49 ± 5% *n* = 2007	12 ± 3% *n* = 479
iPD_OFF_EXP_	20	29 ± 4% *n* = 683	49 ± 5% *n* = 1098	21 ± 4% *n* = 464

Mean percentage ± se of the different response types observed.

*CONT* control, *iPD* idiopathic Parkinson’s disease patients, *ON* ON L-DOPA testing, *OFF* OFF L-DOPA testing, *IMP* implicit condition, *EXP* explicit condition, *n* total number of trials in each category.

In controls, the percentage of early saccades was low in the implicit case (6%) but increased in the explicit one (18%) although the percentage of failed trials remained constant (implicit, 20% → explicit, 19%). In iPD patients ON L-DOPA, the explicit condition was also associated with a larger number of early saccades compared with the implicit one (implicit, 9% → explicit, 20%). The L-DOPA OFF state only modestly altered the proportion of early saccades in the implicit (9% → 12%) and explicit conditions (20% → 21%). A linear mixed-model (LMM) analysis of the percentage of early saccades revealed a significant main effect of the implicit/explicit context (log_10_ transformation of percentages; F[1, 77.395] = 22.341; *p* < 0.001) but no significant effect of group or interaction between factors. Therefore, the percentage of occurrence of early saccades was statistically similar in the different groups and similarly affected by the presence of a temporal cue.

**Fig. 2:**
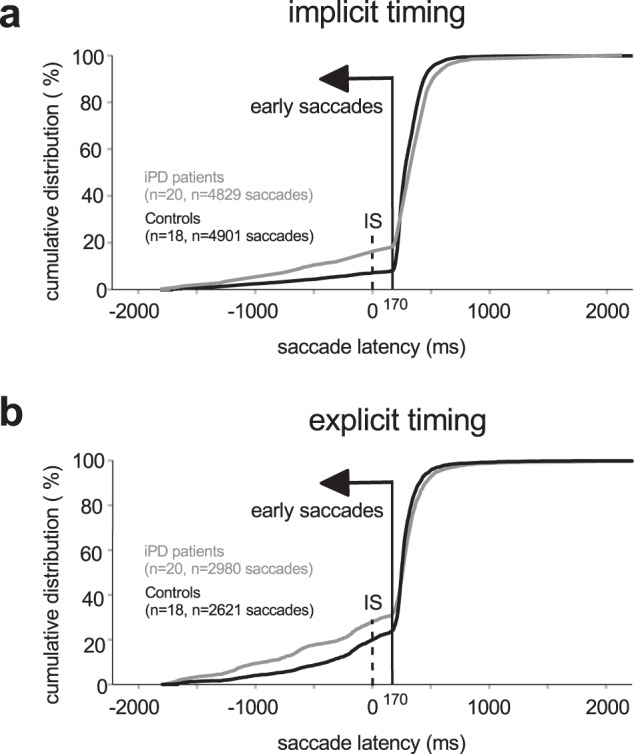
Cumulative saccadic latency histograms. Implicit (**a**) and explicit (**b**) timing conditions. *T*_*ON*_, target (imperative stimulus) onset. Early saccades occur before the inflection point in the distributions (latency < 170 ms, vertical continuous line). Time zero on the X-axis represents the onset of the IS.

Figure 2 shows the cumulative latency distributions for all saccades in the different groups and conditions. A slow increase of the cumulative number of observations can be observed followed by an abrupt slope change. The latency of this transition point (change of slope) was consistently found to be 170 ms in the implicit (Fig. 2a) and explicit cases (Fig. 2b) in both controls and patients. This latency served as a cut-off to separate early saccades (latency ≤ 170 ms) from visually-guided ones (latency > 170 ms). Although some saccades considered as early occurred after the IS had appeared, they were likely planned before visual information was available to the saccadic system.

**Fig. 3:**
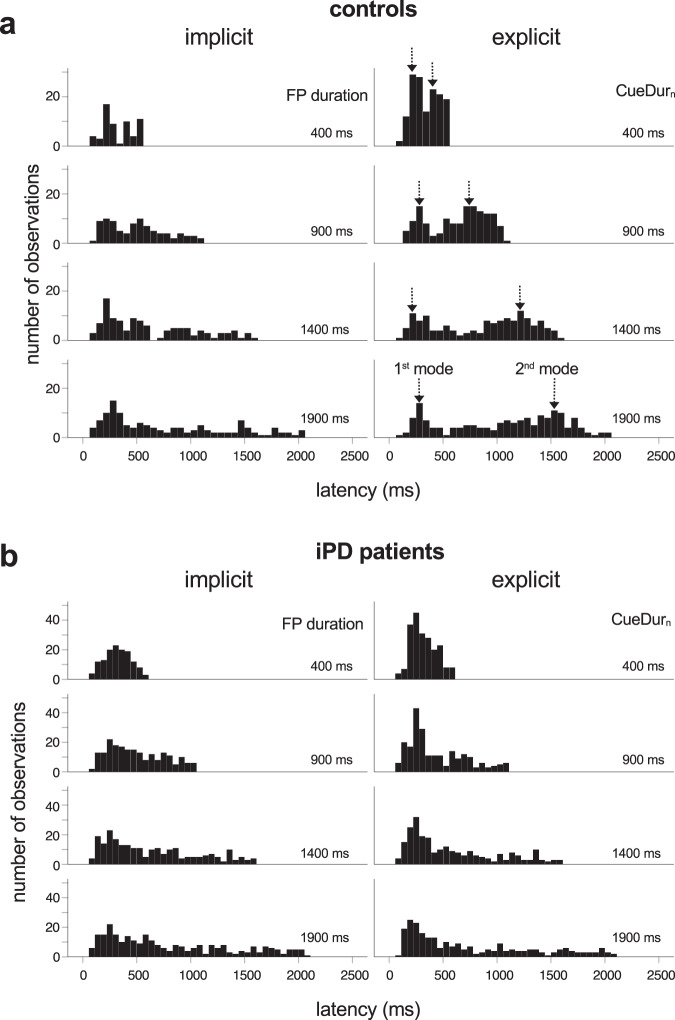
Histograms of latency distributions. Early saccades in controls (**a**) and iPD patients (**b**). Left column: implicit timing; right column: explicit timing. Vertical arrows are centered on the peak of the 1^st^ and 2^nd^ modes of the explicit latency distributions in controls for illustrative purposes. Saccadic latency was measured with respect to the offset of the WS (time ‘zero’ on the X-axis).

**Fig. 4:**
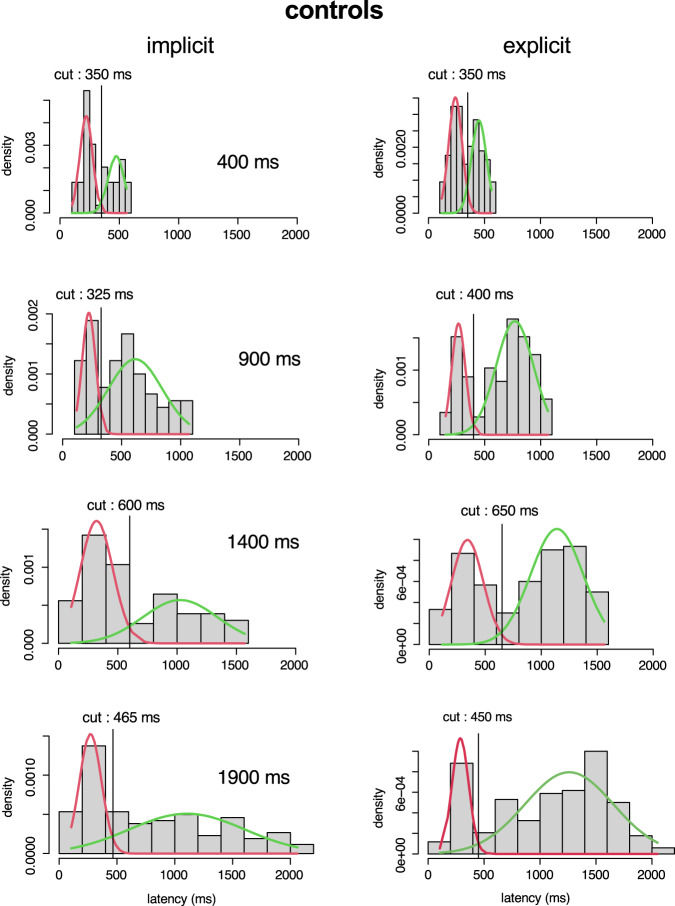
Mixture model analysis. Saccadic latency probability densities in controls in the implicit (left) and explicit conditions (right) in controls as an example. Red curves: 1^st^ mode responses; green curves: 2^nd^ mode responses. The ‘cut’ between 1^st^ and 2^nd^ modes was deterministically placed at the intersection of overlapping Gaussian functions for the different durations tested.

Figure 3 shows latency histograms for early saccades after the cut-off was applied, aligned on the offset of the WS (time zero on the X-axis). In the implicit case (left column in Fig. 3a, b), a large number of saccades occurred approximately 250 ms after the offset of the WS. Thereafter, the occurrence of saccades progressively decayed. In the explicit case (right column in Fig. 3a, b), latency histograms show also an initial peak 250 ms after the offset of the WS (the ‘1^st^ mode’) followed by another one made up of saccades with longer latencies (‘2^nd^ mode’). This bimodality was clearly present in controls in the explicit case but reduced in iPD patients. Moreover, in the explicit case in controls, the number of observations around the 1^st^ mode tended to progressively decrease with increasing cue duration (CueDur_n_) in contrast with observations around the 2^nd^ mode that tended to occur more frequently. These observations suggest that two different stochastic processes could explain observed distributions. Consequently, latency distributions were fitted with 2 Gaussian distributions to create a finite mixture model using an expectation maximization algorithm (‘mixtools’ R package^47^, ‘normalmixEM’). Figure 4 shows the result of this procedure in the implicit (left column) and explicit timing conditions (right column) in controls, as an example. In order to separate the 1^st^ from 2^nd^ mode latencies, a ‘cut’ was defined as the latency of the crossing point between the two Gaussian densities (see Fig. 4, vertical lines). This procedure was applied for each CueDur_n_ (explicit case) and FP duration (implicit case) separately in each group of subjects and experimental conditions and provided a mixing proportion of 1^st^ mode and 2^nd^ mode (see Table 3).

**Table 3:** Mixing proportions of 1^st^ and 2^nd^ mode responses.

FP/CueDur_n_ (ms)	400	900	1400	1900
*CONT*				
Implicit				
1^st^ mode	0.57	0.29	0.55	0.36
2^nd^ mode	0.43	0.71	0.45	0.64
Explicit				
1^st^ mode	0.53	0.26	0.35	0.21
2^nd^ mode	0.47	0.74	0.65	0.79
*iPD*				
Implicit				
1^st^ mode	0.08	0.42	0.40	0.49
2^nd^ mode	0.92	0.58	0.60	0.51
Explicit				
1^st^ mode	0.22	0.53	0.44	0.44
2^nd^ mode	0.78	0.47	0.56	0.56

Controls (CONT) and iPD patients. Each column represents the proportion of responses in each mode ([number of 1st or 2nd mode responses]/[number of 1st mode responses + number of 2nd mode responses]).

**Fig. 5:**
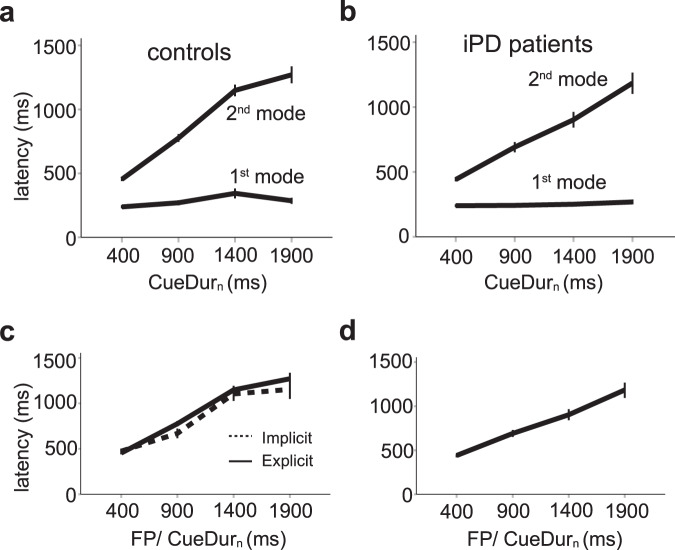
Aggregated data. Upper part: average saccadic latency of 1^st^ (red lines) and 2^nd^ mode responses (blue lines) as a function of CueDur_n_ in controls (**a**) and iPD patients (**b**). Lower: comparison of 2^nd^ mode average latencies in the implicit (*dashed curves*) and explicit conditions (continuous curves) in controls (**c**) and iPD patients (**d**). Error bars: 95% confidence interval of the mean.

In the explicit condition in controls, mixing proportions show that the probability to observe a 1^st^ mode response decreased whereas the opposite trend was observed for 2^nd^ mode responses (Table 3, upper). This transfer of saccadic latencies between response modes was less pronounced in iPD patients (Table 3, lower). Given the likely existence of two independent stochastic processes in the explicit condition, a mixed-model analysis was performed on 1^st^ and 2^nd^ mode latencies separately with CueDur_n_ as within-subject factor (explicit case) and subject group (controls vs iPD) as between-subjects factor. Figure 5a, b shows the relationship between CueDur_n_ and saccadic latency for 1^st^ and 2^nd^ mode responses in controls and iPD patients, respectively. For 1^st^ mode responses, a LMM analysis revealed a significant main effect of CueDur_n_ was found (F[3, 680.476] = 17.113; *p* < 0.001). There was no influence of subject group (F[1, 34.702] = 2.037; *p* = 0.162) but a significant interaction between factors (CueDur_n_ x group; F[3, 680.476] = 11.048; *p* < 0.001). Therefore, CueDur_n_ altered 1^st^ mode latencies differently between groups. A linear regression analysis revealed that the slope of the linear relationship between CueDur_n_ and movement latency was positive and 2.3 times steeper in controls (F[1, 208] = 21.474; *β* = 0.306; *t* = 4.634; *p* < 0.001; *r*^2^ = 0.089) than in iPD patients (F[1, 486] = 8.709; *β* = 0.133; *t* = 2.951; *p* = 0.003; *r*^2^ = 0.016) but effect sizes were small (9% of the variance accounted for in controls and 2% in iPD patients). For 2^nd^ mode responses, a LMM analysis revealed a significant main effect of CueDur_n_ was also found (F[3, 817.598] = 260.221; *p* < 0.001). Here also, there was no main influence of subject group (F[1, 30.428] = 3.350; *p* = 0.077) but a significant interaction between factors was found (CueDur_n_ x group; F[3, 817.598] = 5.274; *p* = 0.001). A linear regression analysis revealed that the slope of the linear relationship between CueDur_n_ and saccade latency was steeper in controls (F[1, 401] = 498.854; *β* = 0.745; *t* = 22.335; *p* < 0.001; *r*^2^ = 0.553) than in iPD patients (F[1, 437] = 283.817; *β* = 0.627; *t* = 16.847; *p* < 0.001; *r*^2^ = 0.392). Effect sizes were large (55% of the variance accounted for in controls and 39% in iPD patients). In iPD patients, there was no significant main effect of L-DOPA treatment or interaction with CueDur_n_ on movement latency in both 1^st^ (LMM, main effect: L-DOPA status: F[1, 469.459] = 4.198; *p* = 0.041; CueDur_n_ x L-DOPA status: F[3, 469.118] = 0.334; *p* = 0.801) and 2^nd^ mode latencies in the explicit case (L-DOPA status: F[1, 428.824] = 0.218; *p* = 0.641; CueDur_n_ x L-DOPA status: F[3, 421.197] = 0.262; *p* = 0.853).

**Fig. 6:**
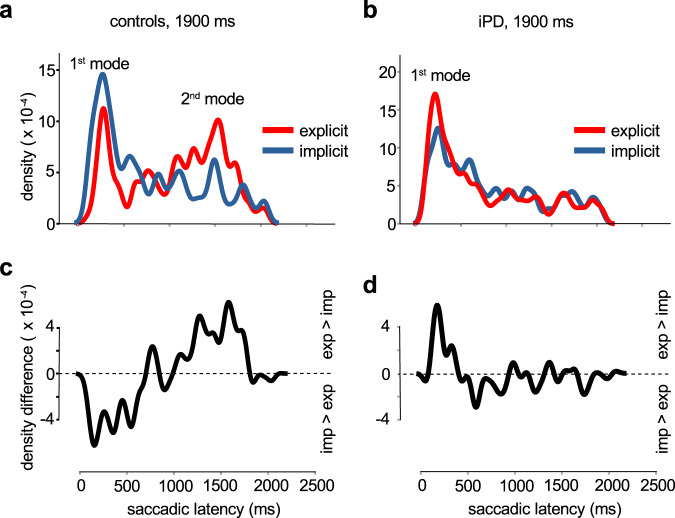
Comparison of probability densities. Upper part: probability densities of saccadic latencies in the explicit (red curve) and implicit (blue curve) conditions for the 1900 ms FP duration in controls (**a**) and iPD patients (**b**). Densities were obtained by convolving latency distributions with a Gaussian kernel of fixed width (40 ms). Lower part: difference of explicit and implicit latency densities for controls (**c**) and iPD patients (**d**).

Figure 5 shows also a direct comparison of saccadic latencies in the implicit and explicit conditions for 2^nd^ mode responses in controls (Fig. 5c) and iPD patients (Fig. 5d). Only a main effect of FP/CueDur_n_ was found in controls (F[3, 593.004] = 176.161; *p* < 0.001) and patients (F[3, 911.584] = 177.675; *p* < 0.001) by LMM. This result shows that the explicit or implicit nature of the task did not play a significant role on *average* saccadic latency. This result was not surprising, given that some 2^nd^ mode responses could occur in the implicit condition due to the influence of the passage of time itself without any source of explicit information. These saccades could be triggered by a weak expectation of the IS, even in the implicit case. This phenomenon could be characterized as the ‘base rate’ of early saccades. Therefore, simply comparing average saccadic latencies did not capture the influence of CueDur_n_. A changing *shape* of the latency distributions could be the main effect of the implicit/explicit conditions. Figure 6a shows probability density functions of saccadic latencies in the implicit (blue curves) and explicit cases (red curves) in controls for FP or CueDur_n_ = 1900 ms.

In controls, 1^st^ mode responses were more likely in the implicit condition and 2^nd^ mode responses more likely in the explicit condition. In iPD patients (Fig. 6b), 1^st^ mode responses were more likely in the explicit condition and 2^nd^ mode responses were equally likely in each condition (base rate of anticipation). This is further illustrated in Fig. 6c (controls) and 6d (patients) which show differences of probability densities (explicit *minus* implicit). In controls, the explicit condition was associated with a decreased probability of 1^st^ mode responses and an increased probability of 2^nd^ mode responses. In iPD patients, the explicit condition was associated with an increased probability of 1^st^ mode responses and a similar 2^nd^ mode probability (‘base rate’ unaltered). The shape of latency distributions in the implicit and explicit conditions were compared in controls and iPD patients for the 4 durations tested using the Kolmogorov–Smirnov Z test (see Table 4). In controls, a significant difference between latency distributions was found except for 400 ms. This duration was probably too short to allow for the emergence of a 2^nd^ mode. In iPD patients, only one significant difference was observed for 900 ms. However, this statistical difference was due to a strong increase of the number of 1^st^ mode saccades in the explicit condition (implicit, *n* = 94; explicit, *n* = 155). In summary, 1^st^ mode saccades were weakly influenced by prior temporal information and were more frequent in iPD patients. This is in contrast to 2^nd^ mode saccades that were strongly influenced by the temporal cue and were more frequent in controls. Therefore, we suggest that latencies in the 1^st^ mode correspond to premature saccades whereas latencies in the 2^nd^ mode of the distribution correspond to temporally-guided genuine anticipatory ones.

**Table 4:** Kolmogorov–Smirnov Z test.

FP/CueDur_n_ (ms)	CONT	iPD
400	0.954 (*p* = 0.322) n_1_ = 59, n_2_ = 148	0.886 (*p* = 0.413) n_1_ = 134, n_2_ = 211
900	2.258 (*p* < 0.001) n_1_ = 90, n_2_ = 145	1.918 (*p* < 0.001) n_1_ = 207, n_2_ = 224
1400	2.151 (*p* < 0.001) n_1_ = 116, n_2_ = 150	1.134 (*p* = 0.153) n_1_ = 243, n_2_ = 243
1900	2.263 (*p* < 0.001) n_1_ = 131, n_2_ = 170	1.099 (*p* = 0.178) n_1_ = 276, n_2_ = 249

Controls (CONT) and iPD patients. n_1_ = sample size implicit condition; n_2_ = sample size explicit condition.

The latency of visually-guided saccades was also compared with LMM between groups and conditions. In controls, average latency was 319 ± 2 ms (*n* = 4465 saccades) in the implicit case and 317 ± 5 ms (*n* = 1987 saccades) in the explicit one. In iPD patients, average saccadic latency was 376 ± 3 ms (*n* = 3914 saccade) in the implicit case and 364 ± 6 ms (*n* = 2026) in the explicit one. There was no significant main effect of the implicit/explicit condition on visually-guided saccade latencies (F[1, 12366.346] = 6.614, *p* = 0.010). Although saccadic latencies were apparently, on average, longer in iPD patients, there was no significant group effect (F[1, 35.822] = 6.402, *p* = 0.016) and no condition x group interaction (F[1, 12366.346] = 0.363, *p* = 0.547). In summary, explicit cueing had statistically little influence on visually-guided eye movements.

### Influence of the context of behavioral states: a micro level analysis

**Fig. 7:**
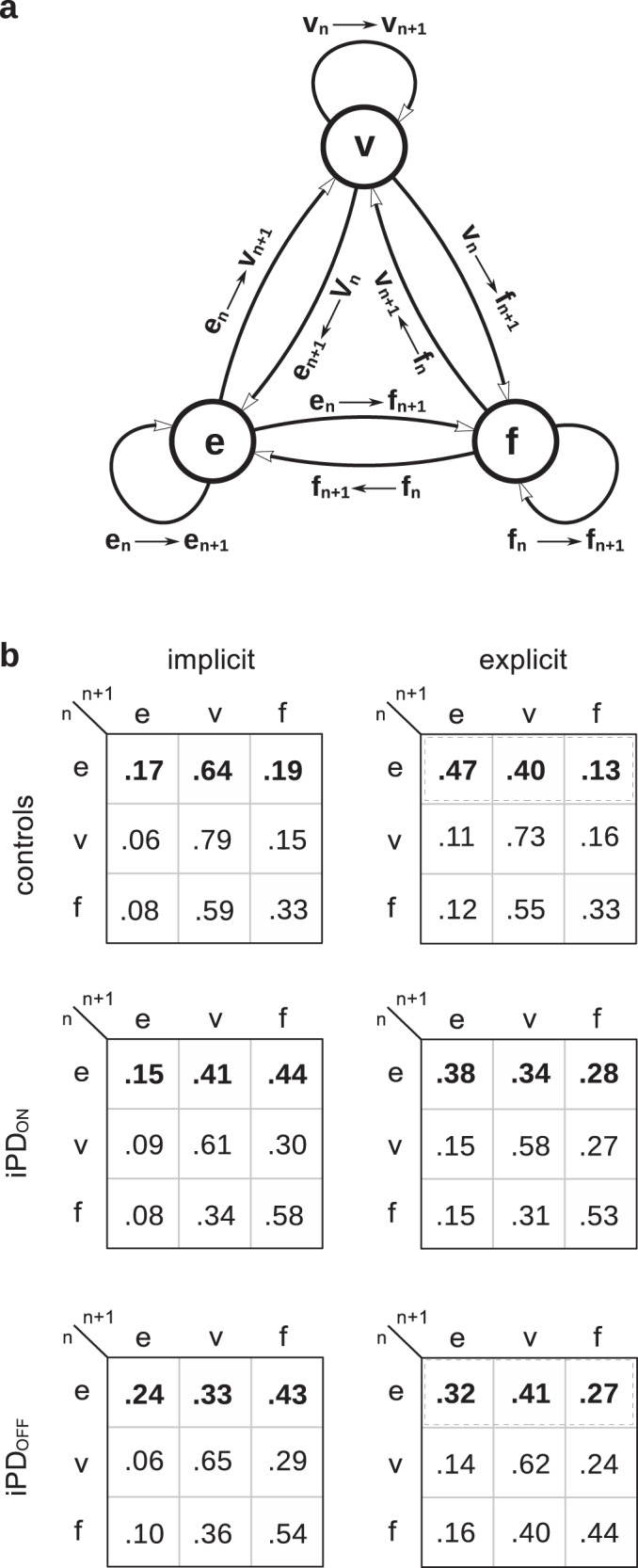
3-state Markov process. **a** Schematic representation of the 3-state Markov process hypothetically underlying sequences of trials in the implicit and explicit conditions for both groups of subjects. Each node represents a given state (‘*e*’: early response; ‘*v*’: visually-guided saccade; ‘*f*’: failed trial). Arrows represent transitions between states in trial ‘n’ and ‘n + 1’. Note that a transition can lead to the same state during trial ‘n’ and trial ‘n + 1’ (looping back arrows). All states were reachable regardless of the starting state forming a communicating class and the chain was irreducible without absorbing state (i.e., impossibility to escape from a state). These states were mutually exclusive (e.g., a trial cannot be e & v) and exhaustive given that all trials belonged to one or the other category. **b** Transition matrices for the different groups and conditions. Dashed rectangles indicate significant differences in the e_n_ row in the explicit condition.

The analysis presented above did not provide any information about the probability of observing either an early response or a visually-guided saccade during a particular trial. To answer to this question, an observable Markov stochastic process analysis was performed in the two groups of subjects and timing conditions^48^. Three observable states were determined: trial ‘n’ could be either a visually-guided response (latency > 170 ms, abb. ‘v’), an early response (latency ≤ 170 ms; ‘e’) or a failure (‘f’). Trials formed a sequence of states within a block of trials (e.g., the sequence ‘e-e-v-v-f-e-v’). This sequence starts in a given state during the first trial (n) and then moves to another state in the next trial (n + 1) with possible states being S = {e, v, f} (Fig. 7a) and the process regenerates itself during succeeding trials.

Formally, the Markov property states:

1 $${{{\mathrm{P}}}}\left( {{{{\mathrm{S}}}}_{{{{\mathrm{n}}}} + 1} = {{{\mathrm{x}}}}_{{{{\mathrm{n}}}} + 1}\left| {{{{\mathrm{S}}}}_{{{\mathrm{n}}}} = {{{\mathrm{x}}}}_{{{\mathrm{n}}}},{{{\mathrm{S}}}}_{{{{\mathrm{n}}}} - 1} = {{{\mathrm{x}}}}_{{{{\mathrm{n}}}} - 1},{{{\mathrm{S}}}}_{{{{\mathrm{n}}}} - 2} = {{{\mathrm{x}}}}_{{{{\mathrm{n}}}} - 2} \ldots .} \right.} \right) = {{{\mathrm{P}}}}\left( {\left. {{{{\mathrm{S}}}}_{{{{\mathrm{n}}}} + 1} = {{{\mathrm{x}}}}_{{{{\mathrm{n}}}} + 1}} \right|{{{\mathrm{S}}}}_{{{\mathrm{n}}}} = {{{\mathrm{x}}}}_{{{\mathrm{n}}}}} \right)$$

with x ∈ {e, v, f}.

**Fig. 8:**
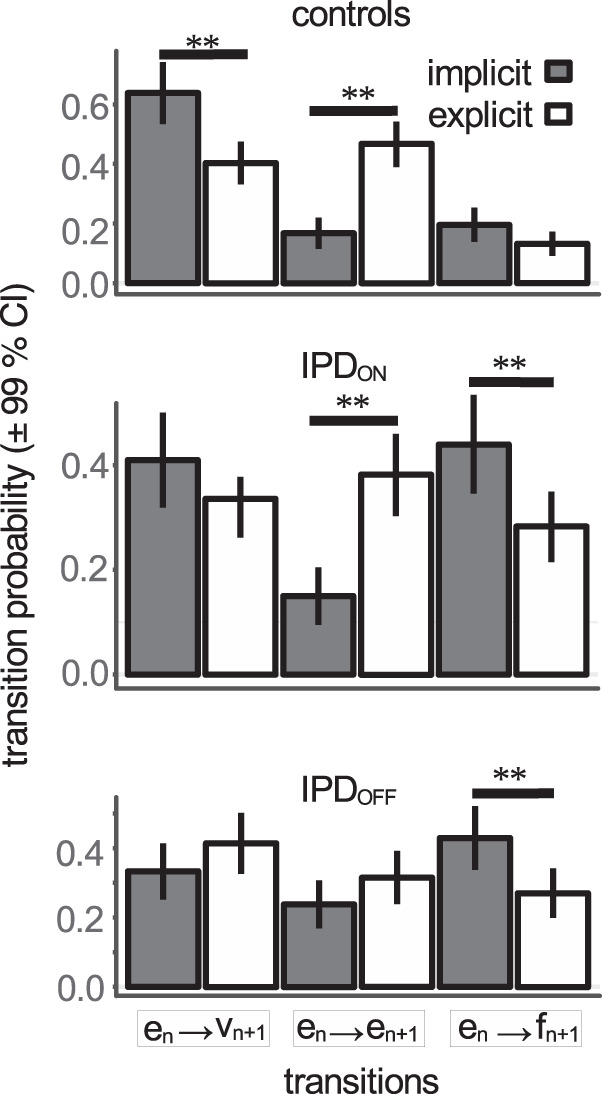
Transition probabilities in the e_n_ row. Implicit (dark bars) and explicit conditions (open bars). Red lines above error bars indicate non-overlapping 99% confidence intervals (i.e., significant difference *p* < 0.01) between the implicit and explicit conditions using the method of Schenker & Gentleman.

The Markov property was tested separately in each subject, condition and group separately (‘verifyMarkovProperty’ function, ‘markovchain’ R package version 0.8.5-3^49,50^). One control subject and two patients did not pass the test and were removed. Response sequences of all subjects were pooled together to estimate the empirical transition matrices for the different experimental conditions and groups (Fig. 7b). Transition probabilities and confidence intervals were computed using a maximum likelihood estimation (MLE, ‘markovchainFit’, *op. cit*.). We found that the behavioral response during trial ‘n’ altered the probability of occurrence of an ‘e’, ‘v’ or ‘f’ response during trial ‘n + 1’ (Supplementary Table 1 provides further details with sample sizes and tests of significance of the difference between probabilities in a given row of the matrices). The preceding trial (n) played a determining role in the occurrence of an e, v or f state in the next trial (n + 1). In the implicit condition in controls, the ‘e_n_ → e_n+1_’ transition was rather infrequent (0.17) compared with an ‘e_n_ → v_n+1_’ transition (0.64). A similar effect was observed in the iPD_ON_ and iPD_OFF_ cases but with an increased probability of failed trials in patients. In the explicit condition, probabilities of transitions in the ‘e_n_’ row were different in controls (χ^2^ [2, 526] = 100.224, *p* < 0.001; dashed red on Fig. 7b). However, a uniform distribution was a better model for the e_n_ row in the ON L-DOPA condition (χ^2^ [2, 410] = 5.624, *p* = 0.06). In the OFF L-DOPA case, the hypothesis of a uniform distribution of state transitions was rejected, as it was in controls (χ^2^ [2, 353] = 10.612; *p* = 0.005). However, this effect was probably due to a further reduction of the probability of an e_n_ → e_n+1_ transition in this group (0.315). Figure 8 shows transition probabilities in the implicit and explicit conditions in the e_n_ row only. In controls and patients ON L-DOPA, the probability of an e_n_ → e_n+1_ transition significantly increased in the explicit case (*p* < 0.01). In both ON and OFF L-DOPA conditions, the number of failed trials significantly decreased in the explicit condition.

**Fig. 9:**
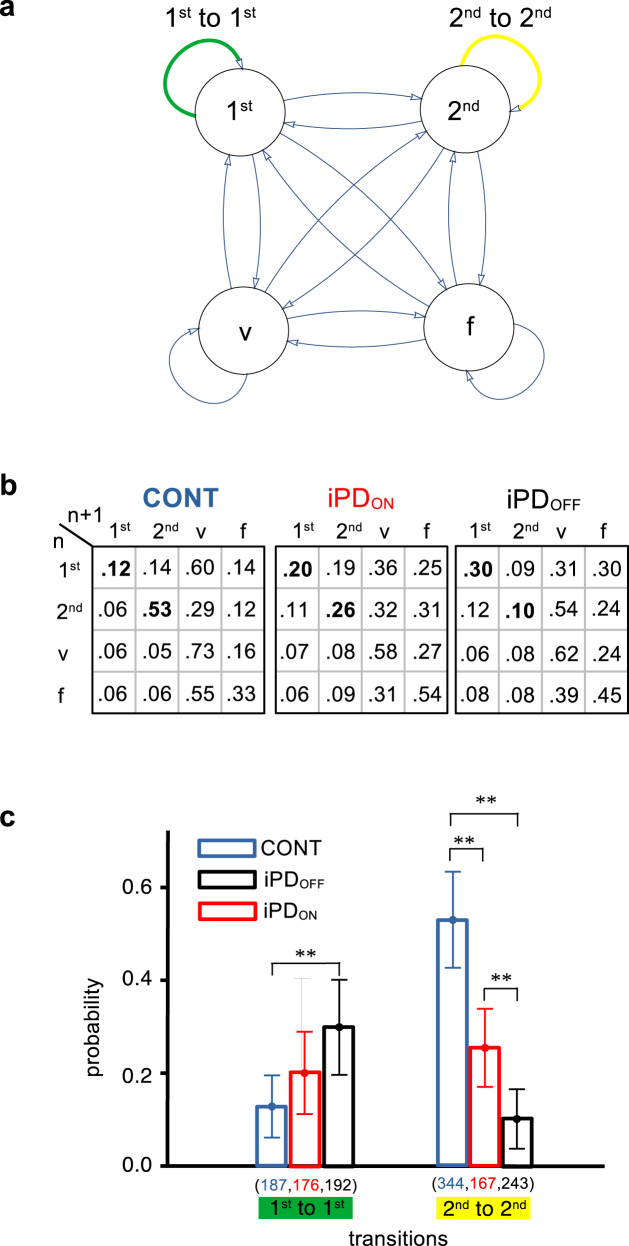
4-state Markov process. **a** Schematic representation of the 4-state Markov process hypothetically underlying sequences of trials in the explicit condition. Each node represents a given state (‘*1*^*st*^’: first mode; ‘*2*^*nd*^’: second mode; ‘*v*’: visually-guided saccade; ‘*f*’: failed trial). Arrows represent transitions between states in trial ‘n’ and ‘n + 1’. The green arrow represents a sequence of two 1^st^ mode responses. The yellow arrow represents a sequence of two 2^nd^ mode responses. **b** Transition matrices for the different groups (iPD or control) and treatments (ON or OFF L-DOPA). Same color code as in **a**. **c** Transition probabilities for 1^st^ to 1^st^ and 2^nd^ to 2^nd^ transitions in the different groups of subjects. Stars indicate significant differences at *p* < 0.003 using the method of Schenker & Gentleman with Bonferroni correction for multiple comparisons (*n* = 3).

The Markov process analysis presented above shows the behavioral context and conditions favorable to the occurrence of early saccades considered indiscriminately. It did not explain which conditions could lead to either a 1^st^ mode premature or a 2^nd^ mode anticipatory saccade during a trial. However, this could determine the shape of the latency densities as presented above on Fig. 6. Therefore, a 4-state observable Markov process could yield a better description. Indeed, the mixture model analysis supports the hypothesis of two independent stochastic processes. However, a 4-state model increases the number of possible transitions from 9 to 16, reducing available sample sizes for the purpose of estimating probabilities. For instance, in the CONT_IMP_ condition, the e_n_ → e_n+1_ transition was observed 65 times in the 3-state model (see Supplementary Table 1a). This sample would have to be further subdivided into four subsamples in a 4-state model (1^st^ → 1^st^, 1^st^ → 2^nd^, 2^nd^ → 1^st^, 2^nd^ → 2^nd^ transitions) with a final sample of 16 saccades. Therefore, the 4-state model was applied only in the explicit condition where samples were larger (e.g., 246 saccades for controls in the e_n_ → e_n+1_ transition; see Supplementary Table 1a). Figure 9a shows the hypothetical underlying Markov process and Fig. 9b transition probabilities. It can be observed that iPD strongly impacted transitions matrices. Sequences of two 1^st^ mode saccades were more frequent in iPD patients (ON: 0.20 and OFF: 0.30) than in controls (0.12). As shown on Fig. 9c, the probability of a sequence of two premature 1^st^ mode saccades increased in the following order: CONT → iPD_ON_ → iPD_OFF_. Sequences of two 2^nd^ mode saccades were frequent in controls (0.53) but decreased in iPD patients (ON: 0.26 and OFF: 0.10). Crucially, transition probabilities in the 4-state model explain the shape of latency densities as the result of two stochastic processes. Indeed, a higher probability of sequences of two 1^st^ mode saccades will ‘shift’ observations towards that mode, particularly in iPD patients. Given that probabilities in a row of the transition matrix must add up to one, increasing sequences of 1^st^ mode saccades occurred at the expense of other type of sequences (e.g., 1^st^ → 2^nd^). Similarly, a higher probability of sequences of 2^nd^ mode saccades will increase the relative number of observations in the second mode, particularly in controls.

In conclusion, the 4-state Markov process analysis shows the transition from anticipation to oculomotor impulsivity induced by iPD, particularly if L-DOPA medication was suspended.

## Discussion

We found that the timing context (implicit or explicit) influenced the probability of observing early saccadic eye movements. However, latency distributions of early saccades were bimodal and could be fitted with two independent Gaussian distributions representing premature (1^st^ mode) and anticipatory saccades (2^nd^ mode), respectively. First mode saccades were weakly influenced by the temporal information provided in contrast to 2^nd^ mode saccades. In controls, changing from the implicit to the explicit timing condition evoked a decrease of 1^st^ mode saccades and an increase of 2^nd^ mode ones. In iPD patients, the same alteration of the temporal context caused an increase of 1^st^ mode saccades. A Markov process analysis with 3 observable states revealed an increase in the probability of an e_n_ → e_n+1_ transition in the explicit case compared to the implicit one in both controls and iPD_ON_ patients. In iPD_OFF_ patients, implicit and explicit transitions in the e_n_ row did not differ.

The 4-state Markov process analysis revealed that sequences of 2^nd^ mode anticipatory saccades were more likely in controls. In contrast, the probability of having sequences of 1^st^ mode premature saccades was higher in iPD and increased with interruption of L-DOPA medication. This observation suggests that a hypothetical L-DOPA overdose cannot explain the observed increase of 1^st^ mode saccades. On the contrary, L-DOPA medication reduced premature saccades and increased anticipatory ones. Therefore, we suggest that the increased probability of 1^st^ to 1^st^ transitions (premature to premature) in iPD could be an *oculo*motor sign of impulsivity. Impulsivity is a multifaceted concept with both cognitive and motor aspects that is usually assessed using questionnaires like the Barratt Impulsiveness scale^51^. Although slowness of movement execution and initiation are characteristics of iPD, impulsivity could sometimes be exacerbated causing more premature movements or decisions^52^, particularly if patients are confronted with difficult choices in the temporal domain^53^. If a motor or behavioral response is not inhibited when inappropriate, then it becomes a sign of impulsivity.

In iPD, the uniformity of the distribution of ‘n + 1’ states in the e_n_ row ON L-DOPA reflects an increased behavioral independence of trials that was even stronger OFF L-DOPA. This independence of successive states could reflect a decreased level of cognitive control. In the domain of gait control, stride duration during prolonged periods is auto-correlated in healthy subjects. This is often quantified using a long-range autocorrelation computation^54^ that is reduced in iPD, suggesting independence of successive strides^55^. The alteration of the P(e_n_ → e_n+1_) transitions in iPD observed here could reflect a similar impact of basal ganglia dysfunction on the integration of sequences of motor responses in a given context.

Eye movements are impacted by iPD^56–59^ and oculomotor anticipation is reduced^60–62^. The increase in latency of visually-guided saccades is usually explained by an overactive inhibition of the superior colliculus (SC) by the substantia nigra pars reticulata (SNr) resulting in oculomotor bradykinesia/akinesia^63,64^. We suggest that this increased pathological inhibition could primarily affect anticipatory saccades but not premature ones. Anticipatory saccades could rely on a network of cortical and subcortical brain areas involving the SC and partly over-inhibited by the SNr. However, premature saccades could be evoked by the disappearance of the WS, causing a transient activation of the oculomotor system that should normally remain inhibited due to task demands. This transient could be enough to open the ‘gate’, perhaps bypassing the BG/SC, and allowing a premature movement to occur. This bypass could occur more frequently in iPD where a failure to inhibit automatic visuomotor responses has been observed^65^.

Results presented here suggest that 1^st^ and 2^nd^ mode saccades are the output of two independent stochastic processes^66^ and that Parkinson’s disease could result in a shift of their relative contribution, leading to more premature responses and less temporal anticipation. This shifting balance could potentially affect motor control in general beyond the specificities of eye movements.

## Methods

### Ethics

Experiments were conducted in agreement with the local Ethics committee and approved by the CERES, « Conseil d’évaluation éthique pour les recherches en santé » of the University Paris Descartes, France (IRB number 20122800001072). The study referred to as ‘PEDUPARK’ has been registered with clinicaltrials.gov (NCT02126475). https://clinicaltrials.gov/ct2/show/NCT02126475?term=NCT02126475&draw=2&rank=1. Participants provided their informed written consent to take part to this study. This consent procedure was approved by the Ethics Committee. Data is available to participants upon written request.

### Participants

All patients fulfilled the UKPDSBB criteria^67^. Control subjects and patients had normal or corrected to normal vision. The L-DOPA equivalent daily dose was computed using the procedure described in ref. ^68^. Patients of the present study were assessed using the Hoehn and Yahr scale^69^ and the motor part of the Unified Parkinson’s disease Rating Scale^70^ (UPDRS part III; see Table 1). The Starkstein scale was used to measure apathy^71^ and the Montreal Cognitive Assessment (MoCA) was used to assess cognitive impairment^72–74^. Subjects with a MoCA score of 25 or below were excluded. All patients were taking medication to control their symptoms (levodopa and/or a dopaminergic agonist). The L-DOPA ‘OFF’ state was characterized by an overnight withdrawal of L-DOPA medication.

### Apparatus

Subjects sat in darkness facing a screen which presented stimuli at a frequency of 60 Hz. An EyeLink 1000 infrared eye tracking system (SR Research, Mississauga, Ontario) was used to record movements of the right eye at 1 KHz. All experiments were run with a software based on a real time Linux kernel (Xenomaï^75^). Saccades were detected offline in MATLAB (MathWorks, Natic, MA) with a velocity threshold of 30 deg/s. In each subject, a total of ~200 experimental trials were collected.

In the implicit timing task, each trial started with an initial fixation period of a small cross (0.7 deg) appearing on the computer screen for a randomized duration (850 ± 100 ms; see ‘X’ on Fig. 1a). At the end of this fixation period two empty ‘boxes’ appeared on the screen (1.4 × 1.4 deg), one in the center and one at a 9 deg eccentric position randomly to the right or to the left randomly. Afterward, a square warning stimulus (WS; 1.4 × 1.4 deg) was flashed in the central box for 50 ms. When the WS was turned off, it indicated to subjects the beginning of the foreperiod (‘FP’). Subjects were required to hold on fixation of the central box until the imperative stimulus (IS; 1.4 × 1.4 deg) was briefly presented for 50 ms in the eccentric box. The FP could take one of 4 different values with the same probability: 400 or 900 or 1400 or 1900 ms. Subjects were asked to wait until IS appearance to make a visually-guided saccade. The implicit timing experiment was executed during the first experimental session.

In the explicit timing task, each trial started with the same initial fixation period (Fig. 1b). However, this period was followed by a visual cue (a red disk, 2 deg diameter) presented at the center of the screen for a duration lasting either 400 or 900 or 1400 or 1900 ms selected randomly with the same probability (cue duration, ‘CueDur_n_’). At the end of cue presentation, the sequence of events was the same as in the implicit condition. The FP lasted for the same duration as the visual cue previously presented and according to the same probability distribution as in the implicit case. The explicit timing experiment was executed during the second experimental session. Subjects and patients were tested twice with one week between experimental sessions. Half the participants suspended L-DOPA intake before the first experimental session and the other half suspended medication before the second one.

### Statistical analyses

A repeated measures linear mixed model approach (LMM) was used to analyze saccadic latencies. In this model, subject identity was used as a random factor to account for the influence of uncontrolled between-subject variability. Each saccadic latency measured for each subject was a data point. This approach is more selective to test experimental effects and interactions and more robust to normality violations and missing data (e.g., no saccade triggered) than standard ANOVA^76–78^. The number of degrees of freedom (df) was estimated using the Satterthwaite algorithm calculated by the MIXED algorithm in SPSS 25 (SPSS Inc., Chicago, IL, United States). Effect sizes were estimated using the coefficient of determination *r*^2^ (variance accounted for by the model). Additional analyzes were performed using R Studio^79^. Significance of observed effects was tested using the F-statistics and α for all analyses was set to 0.01. Comparisons based on confidence intervals of transition probabilities in the Markov process analysis were analyzed using the method described by Schenker and Gentleman^80^ with *α* = 0.01. This method requires to build the 99% confidence interval of the difference between selected transition probabilities and to determine whether it contained zero (no significant difference) or not (significant difference).

## Supplementary information

Supplementary Table 1: Transition probabilities

## Data Availability

The datasets generated during and/or analyzed during the current study are available from the corresponding author on reasonable request.
