# Supplementary material for: From anticipation to impulsivity in Parkinson’s disease
Source: NPJ Parkinsons Dis. 2022 Oct 3;8:125. doi: 10.1038/s41531-022-00393-w (PMC9527232; doi:10.1038/s41531-022-00393-w)
Supplement: Supplementary file 1 — Supplementary Table 1: Transition probabilities [file 41531_2022_393_MOESM1_ESM.pdf]

Supplementary material

a

**Condition**	**Trial**				
**CONT** **IMP**	n \ n+1	v_{n+1}	e_{n+1}	f_{n+1}	**Chi-square test**
	v_n	3240/4088 0.793 [0.757 – 0.829]	240/4088 0.059 [0.049 – 0.069]	608/4088 0.149 [0.133 – 0.164]	χ^2 (2, 4088) = 3929.268, p<0.001
	e_n	244/382 .638 [0.533 – 0.742]	**65/382** **.167** [0.114 – 0.220]	73/382 0.195 [0.138 – 0.253]	χ^2 (2, 382) = 160.592, p<0.001
	f_n	602/1019 .591 [0.529 – 0.653]	84/1019 .083 [0.059 – 0.106]	333/1019 .326 [0.280 – 0.373]	χ^2 (2, 1019) = 395.178, p<0.001
**CONT** **EXP**					
	v_n	1373/1883 0.730 [0.680 – 0.781]	213/1883 0.112 [0.092 – 0.132]	297/1883 0.157 [0.134 – 0.181]	χ^2 (2, 1883) = 1333.209, p<0.001
	e_n	211/526 0.403 [0.331 – 0.474]	**246/526** **0.467** [0.389 – 0.542]	69/526 0.132 [0.091 – 0.173]	χ^2 (2, 526) = 100.224, p<0.001
	f_n	305/549 0.549 [0.467 – 0.630]	65/549 0.123 [0.084 – 0.162]	179/549 0.328 [0.265 – 0.392]	χ^2 (2, 549) = 157.508, p<0.001

b

**Condition**	**Trial**				
**PD_ON** **IMP**	n \ n+1	v_{n+1}	e_{n+1}	f_{n+1}	**Chi-square test**
	v_n	1107/1813 0.611 [0.564 – 0.659]	153/1813 0.085 [0.067 – 0.102]	553/1813 0.304 [0.271 – 0.338]	χ^2 (2, 1813) = 759.532, p<0.001
	e_n	136/329 0.410 [0.319 – 0.501]	**49/329** **0.150** [0.095 – 0.205]	144/329 0.440 [0.346 – 0.535]	χ^2 (2, 329) = 50.632, p<0.001
	f_n	566/1661 0.341	125/1661 0.075 [0.058 – 0.092]	970/1661 0.584	χ^2 (2, 1661) = 645.227, p<0.001

		[0.304 – 0.378]		[0.535 – 0.632]	
**PD_ON EXP**					
	v_n	536/917<br>0.584<br>[0.519 – 0.649]	138/917<br>0.151<br>[0.118 – 0.184]	243/917<br>0.266<br>[0.222 – 0.309]	χ^2 (2, 917) = 278.384, p<0.001
	e_n	139/410<br>0.336<br>[0.262 – 0.409]	**155/410**<br>**0.382**<br>[0.303 – 0.460]	116/410<br>0.283<br>[0.215 – 0.350]	χ^2 (2, 410) = 5.624, p=0.06
	f_n	240/776<br>0.312<br>[0.260 – 0.364]	122/776<br>0.154<br>[0.118 – 0.190]	414/776<br>0.534<br>[0.467 – 0.602]	χ^2 (2, 776) = 166.835, p<0.001

c

**Condition**	**Trial**				
**PD_OFF$_{IMP}$**	n+1 / n	v_{n+1}	e_{n+1}	f_{n+1}	**Chi-square test**
	v_n	1200/1853<br>0.649<br>[0.600 – 0.697]	109/1853<br>0.058<br>[0.044 – 0.073]	544/1853<br>0.293<br>[0.261 – 0.325]	χ^2 (2, 1853) = 976.709 p<0.001
	e_n	113/339<br>0.333<br>[0.252 – 0.414]	**80/339**<br>**0.238**<br>[0.169 – 0.307]	146/339<br>0.429<br>[0.337 – 0.521]	χ^2 (2, 339) = 19.274 p<0.001
	f_n	540/1476<br>0.365<br>[0.324 – 0.405]	147/1476<br>0.100<br>[0.079 – 0.121]	789/1476<br>0.535<br>[0.486 – 0.584]	χ^2 (2, 1476) = 425.890 p<0.001
**PD_OFF$_{EXP}$**				617/2007=0.31	
	v_n	643/1034<br>0.619<br>[0.556 – 0.682]	145/1034<br>0.141<br>0.111 – 0.171]	246/1034<br>0.240<br>0.200 – 0.279]	χ^2 (2, 1034) = 403.733 p<0.001
	e_n	145/353<br>0.414<br>[0.326 – 0.502]	**112/353**<br>**0.315**<br>[0.239 – 0.392]	96/353<br>0.270<br>[0.199 – 0.342]	χ^2 (2, 353) = 10.612 p = 0.005
	f_n	246/620<br>0.399<br>[0.333 – 0.464]	99/620<br>0.157<br>[0.116 – 0.198]	275/620<br>0.444<br>[0.375 – 0.513]	χ^2 (2, 620) = 86.171 p<0.001

Supplementary Table 1: Transition probabilities Number of observations and transitions probabilities in the implicit and explicit timing conditions in control subjects (**a**) and patients

(**b**, **c**). The first line in each cell indicates the number of saccades in each transition divided by the total number of saccades in a row. The second number gives the value of the transition probability and the 99% confidence interval. χ^2 test: testing the hypothesis that all transition probabilities should be equal in a given row of the matrix (e.g. H_0: $P[e_n \text{ -> } e_{n+1}] = P[e_n \text{ -> } v_{n+1}] = P[e_n \text{ -> } f_{n+1}]$; H_1 : at least one transition probability is different, with $p<0.01$). *CONT*, controls; *PD_ON*, patient testing after L-DOPA intake; *PD_OFF*, patient testing before L-DOPA intake (see Methods for details).
